# Supplementary material for: Generation of scalable cancer models by combining AAV-intron-trap, CRISPR/Cas9, and inducible Cre-recombinase
Source: Commun Biol. 2021 Oct 13;4:1184. doi: 10.1038/s42003-021-02690-1 (PMC8514589; doi:10.1038/s42003-021-02690-1)
Supplement: Supplementary file 2 — Supplementary information [file 42003_2021_2690_MOESM2_ESM.pdf]

SUPPLEMENTARY INFORMATION

Generation of Scalable Cancer Models by Combining AAV-intron-trap, CRISPR/Cas9, and Inducible Cre-recombinase

Authors: Prajwal Boddu; Abhishek Gupta; Jung-Sik Kim; Karla Neugebauer; Todd Waldman; Manoj Pillai

Contents:

Supplemental Figures 1-5……………………………………………………2
Supplemental Tables 1-3……………………………………………………7
Original western and PCR blot images……………………………………10

Figure S1

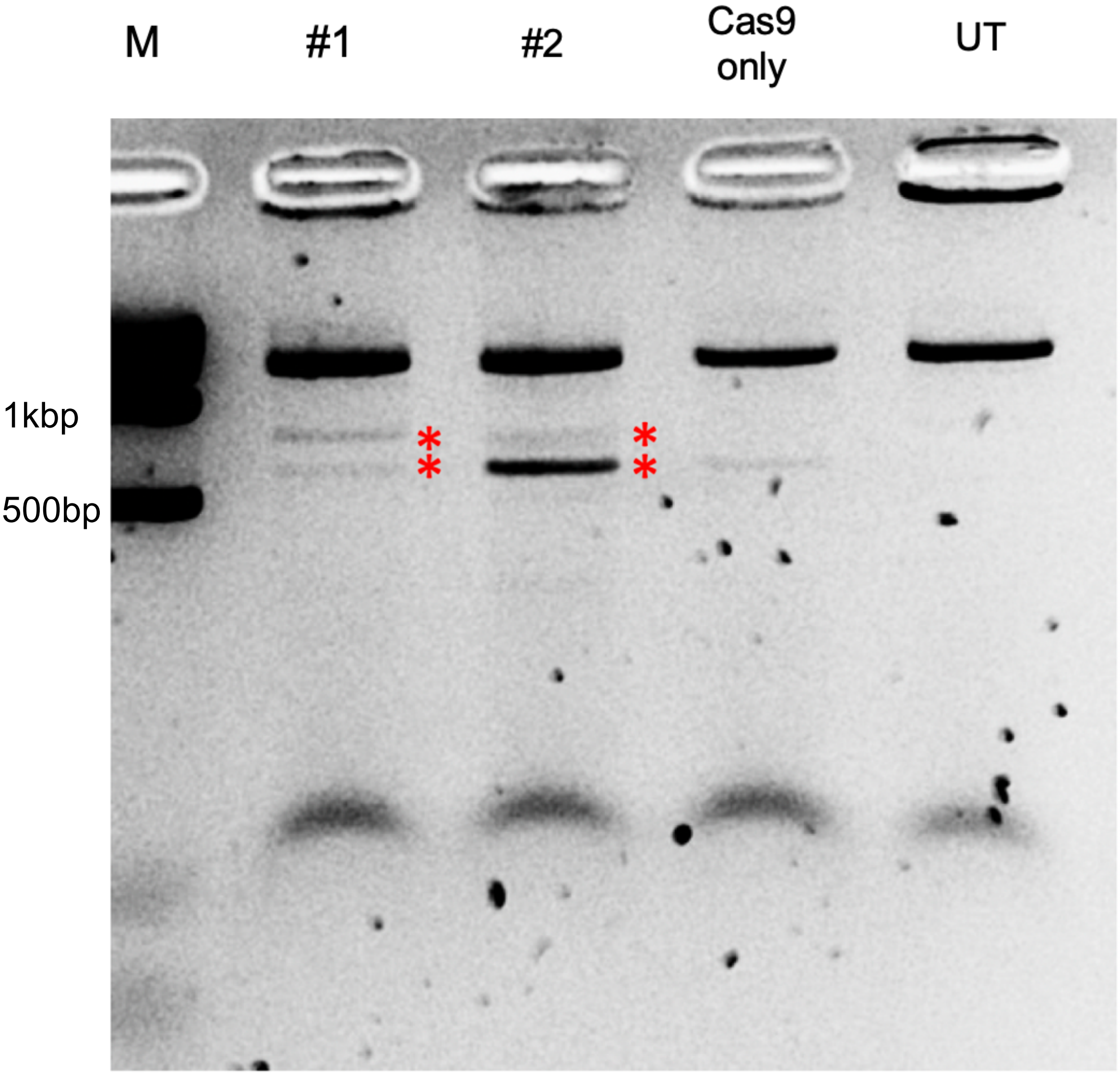

Targeted mutagenesis of SF3B1 detected by the T7EI assay. Four sgRNAs are used in the assay. Asterisks indicate the expected cleavage products/bands. sgRNAs # 1 and # 2 selected for targeted mutagenesis. M-Marker, UT-untransfected

Figure S2

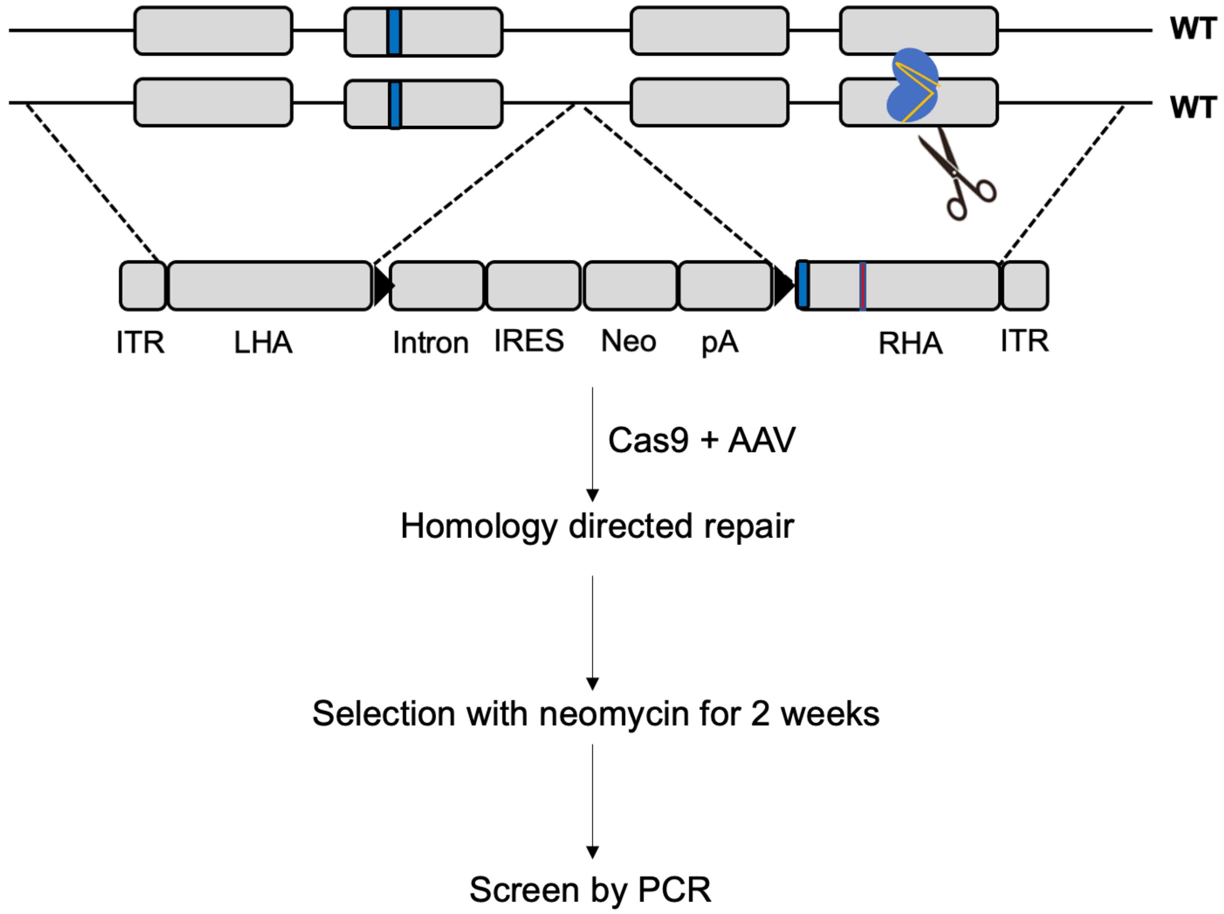

Schematic of genomic SF3B1 locus and AAV vector is shown. AAV vector consists of the left homology arm (LHA); neomycin cassette with IRES, poly(A) termination signal; and a Right Homology Arm (RHA) with the K700E mutation (shown in magenta) and an extra 20 bp from upstream exons (shown in blue) added to its beginning, for PCR screening; LoxP sites (shown by black triangles) were placed, as indicated, to excise out the neomycin resistance cassette after recombination is confirmed.

Figure S3

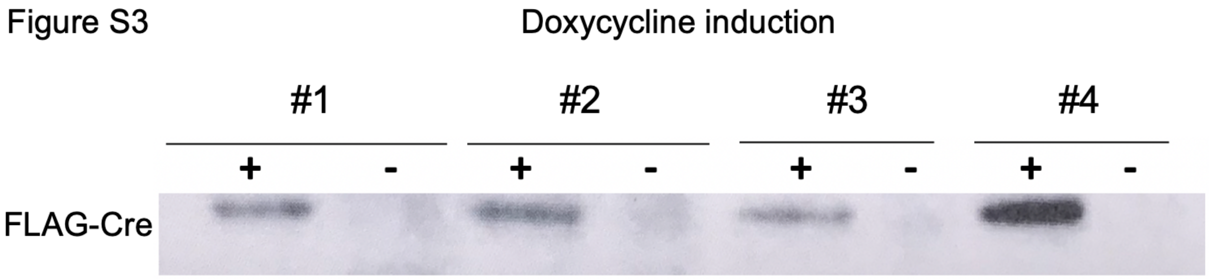

Western blot-based confirmation of expression of the FLAG-tagged Cre-recombinase protein (37kDa). Depicted is the western blot with FLAG antibodies on lysates from 4 parental (*SF3B1*$^{wt/K700E}$) clones, 2 days post-induction with doxycycline.

Figure S4

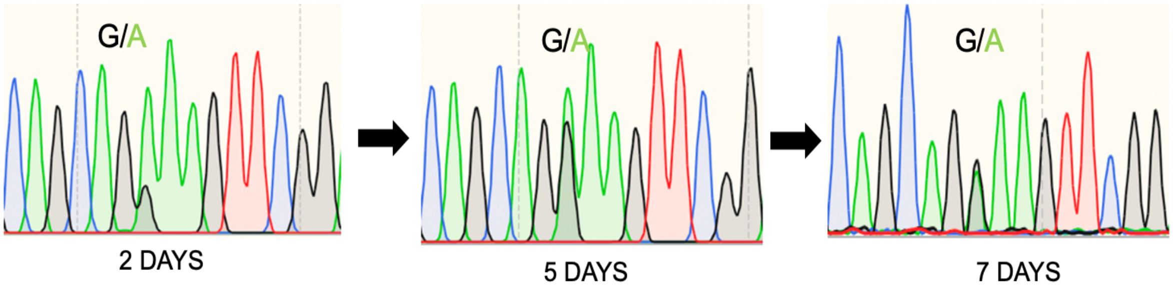

cDNA sequence Sanger chromatograms in K562 isogenic cell line post doxycycline induction. Depicted are chromatograms demonstrating an increasing mutant signal peak over time, after doxycycline induction, reflecting gradual temporal activation of the mutant allele in the sample populations.

Figure S5

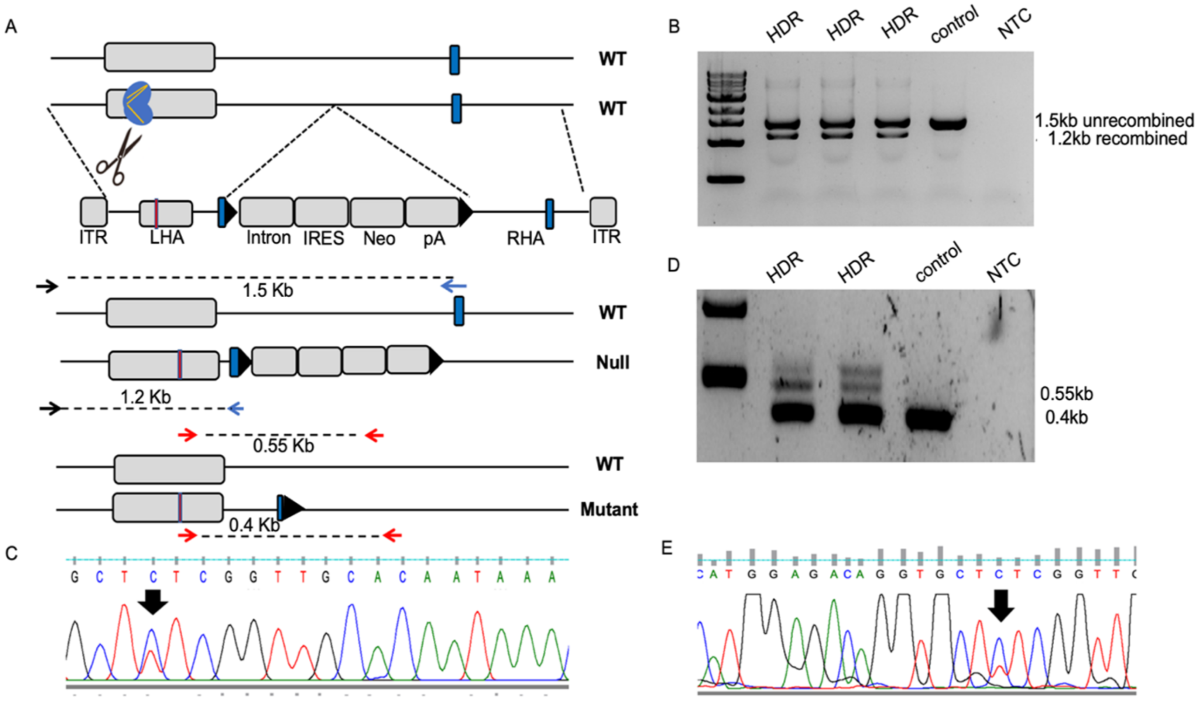

Schematic overview of single allele editing of *U2AF1* locus using CRISPR/Cas9 and rAAV. (a) Schematic of 'In-Out' PCR screening strategy of clones after single allele recombination before Cre-recombinase (black/blue arrows) and after Cre-recombinase (red arrows) activation is shown. (b) PCR analysis of genomic DNA isolated from unedited and edited K562 cells, that underwent recombination (using screening primers denoted by black and blue arrows in the middle panel of figure S5a). (c) Confirmation of successful single allele S34F mutation by Sanger sequencing of genomic DNA. (d) PCR analysis of genomic DNA from edited K562 cells post Cre recombinase activation (using primers denoted by red arrows in the lower panel of figure S5a). (e) Sanger sequencing of cDNA from K562 isogenic cell line sample (2 days post-induction) confirming mutant *U2AF1* mRNA expression (black arrow). HDR- homology directed repair, NTC-no template control.

Table S1: Cloning and screening primers for SF3B1 gene locus editing

Primer name/description	Primer Sequence
LHA forward (F) primer	5’ACCGGTGTGCATAAGGTTTGTTCTCC3’
LHA reverse (R) primer	5’GAGCTCCCAGTCTGGGCAACATAG3’
RHA F primer	5’ACCATCGATACTCAAACCTTTGGGCTCAAG3’
RHA R primer	5’CACGTCGACACCATCTGTCCCACAACAC3’
SDM F to insert screening primer in RHA	5’ACCGCTATTGACTCAAACCTTTGGGCTC3’
SDM R to insert screening primer in RHA	5’TCAATGACCAATCGATAAGCTTGATATCGAATTC3’
SDM F primer for K700E	5’TGAGCAGCAGGAAGTTCGGAC3’
SDM R primer for K700E	5’ TCCACAAGACCTACAAAACC 3’
Pre-Cre F screening primer	5’TGGTCATTGAACCGCTATTG3’
Pre-Cre R screening primer	5’CTGTGCTGCCAGAAGTGTTT3’
Post-Cre F screening primer	5’ACCAACTCATGACTGTCCTTTCT3’
Post-Cre R screening primer	5’CTCCCCAAATCAGTAGCCCA3’
F primer for cDNA sequencing	5’AGGCTGCTGGTCTGGCTACT3’
R primer for cDNA sequencing	5’ACACCATCTGTCCCACAACACTG3’

Table S2: sgRNA and T7EI template primers

Primer name/description	Primer sequence
sgRNA # 1	5’AAGCTAATACGACTCACTATA***G***TGGATGAGCAGCAGAAAGTTG TTTTAGAGCTAGAA3’
sgRNA # 2	5’AAGCTAATACGACTCACTATA***G***TGGATGAGCAGCAGAAAGTTG TTTTAGAGCTAGAA3’
F primer for T7EI assay	5’AGGCTGCTGGTCTGGCTACT3’
R primer for T7EI assay	5’ACACCATCTGTCCCACAACACTG3’

Table S3: Titration of rAAV

qPCR Library Quantification (https://nebiocalculator.neb.com/#!/qPCRGen)--

Summary

Standard Curve	Libraries	
Efficiency: 101.78 % R^2: 0.993 slope: -3.28	SF3B1 w/ DNAse	596713 copies.
	S34F-1 w/DNAse	7371893 copies.
	SF3b1 w/o DNAse	3555025 copies.
	S34F w/o DNAse	18948730 copies.

Detailed Input and Results

Standards

y = 39.13 -3.28x

R^2 = 0.993

Conc. (pM)	C_q1	C_q2	C_q3
100000000	13.38	13.49	13.27
10000000	15.7	15.76	15.63
1000000	19.22	19.18	19.26
100000	22.5	22.38	22.63
10000	26.39	26.41	26.36

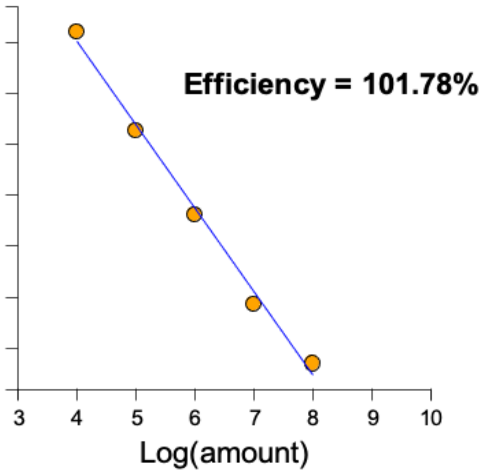

SF3B1K700E w/ DNAse. 596713 copies

Dilution (1:x)	C_q1	C_q2	C_q3	Avg. C_q
10	25.32	27.56		26.44
50	25.22	25.27		25.25
250	27.67	27.63		27.65
1250	30.21	30.16		30.18

U2AF1S34F w/Dnase 7371893 copies

Dilution (1:x)	C_q1	C_q2	C_q3	Avg. C_q
10	21.64	20.77		21.20
50	21.92	21.85		21.89
250	24.29	24.16		24.23
1250	26.53	26.5		26.52

SF3B1K700E W/O DNAse 3555025 copies

Dilution (1:x)	C_q1	C_q2	C_q3	Avg. C_q
10	20.86	20.68		20.77
50	23.19	23.11		23.15
250	25.65	25.42		25.54
1250	28.04	28		28.02

U2AF1S34F W/O DNAse 18948730 copies

Dilution (1:x)	C_q1	C_q2	C_q3	Avg. C_q
10	18.6	18.65		18.63
50	20.75	20.75		20.75
250	23.12	23.05		23.09
1250	25.5	25.42		25.46

Titration of rAAV for *SF3B1*K700E and *U2AF1*S34F performed using NEB online qPCR quantification bio calculator tool (https://nebiocalculator.neb.com/#!/qPCRGen). A standard curve was obtained using the pAAV-SEPT purified plasmid by using serial 10x dilutions of 10^8 to 10^4 copies. The viral genomic physical titer was determined for SF3B1 K700E and U2AF1 S34F rAAV viruses, with and without DNAse1 treatment. qPCR reactions of the test samples performed in technical duplicates

ORIGINAL WESTERN AND GEL BLOT IMAGES:

FIGURE 4b:

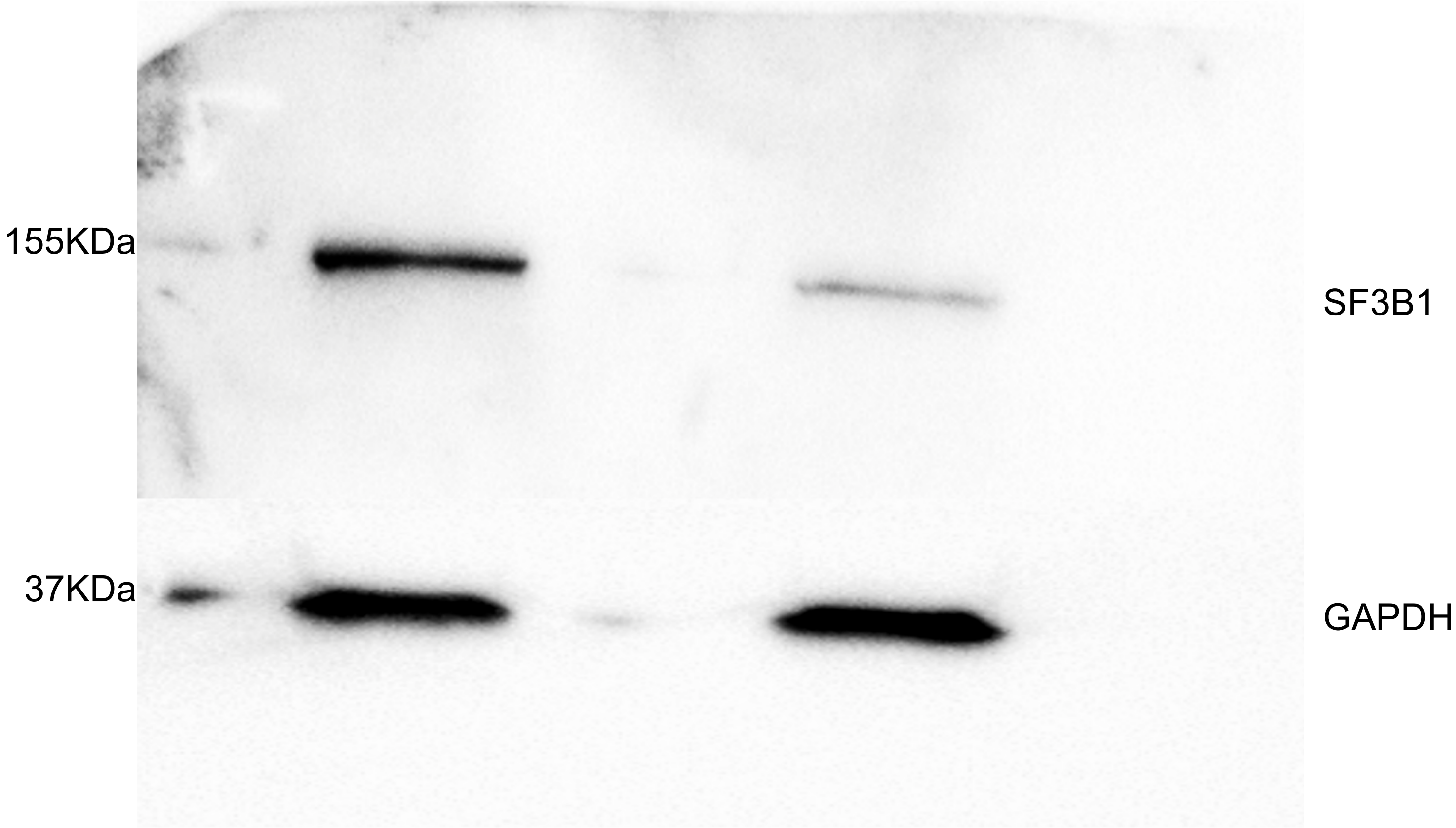

Western Blot-based confirmation of expression of the mutant *SF3B1* allele. Depicted is the western blot with SF3B1 antibodies on lysates from parental (*SF3B1*$^{wt/null}$) clone and cells 10 days post-induction (*SF3B1*$^{wt/K700E}$). The induction of mutant *SF3B1* allele leading to expression of mutant SF3B1 protein (same size as the wild type SF3B1 protein) doubles the SF3B1 protein expression as compared to the pre-induced sample.

FIGURE S3:

37KDa

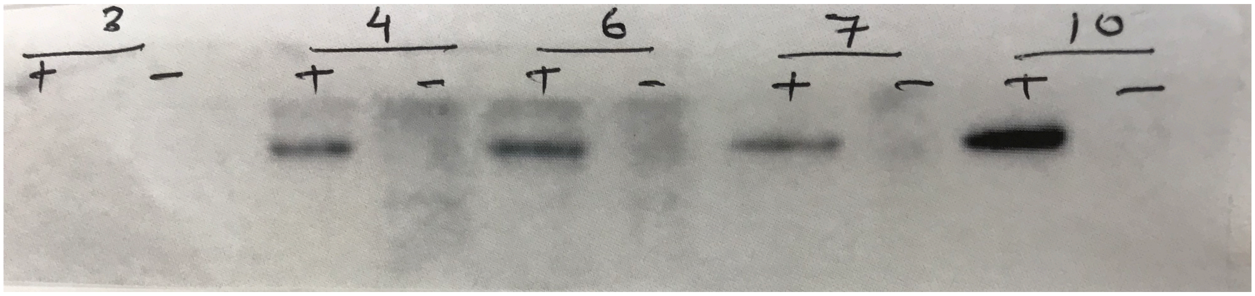

Western blot-based confirmation of expression of the FLAG-tagged Cre-recombinase protein (37kDa). Depicted is the western blot with FLAG antibody on lysates from 5 parental (*SF3B1*$^{wt/K700E}$) clones, 2 days post-induction with doxycycline. Four of five clones (# 4, 6, 7, 10) demonstrated expression of FLAG-tagged Cre-recombinase with doxycycline (+) while clone # 3 did not express the FLAG-Cre protein; as expected, none of the clones express the FLAG-Cre protein in the absence of doxycycline (-). Clones # 4, 6, 7, 10 correspond to clones # 1, 2, 3, 4, respectively, in supplemental Figure S3.

FIGURE 4c:

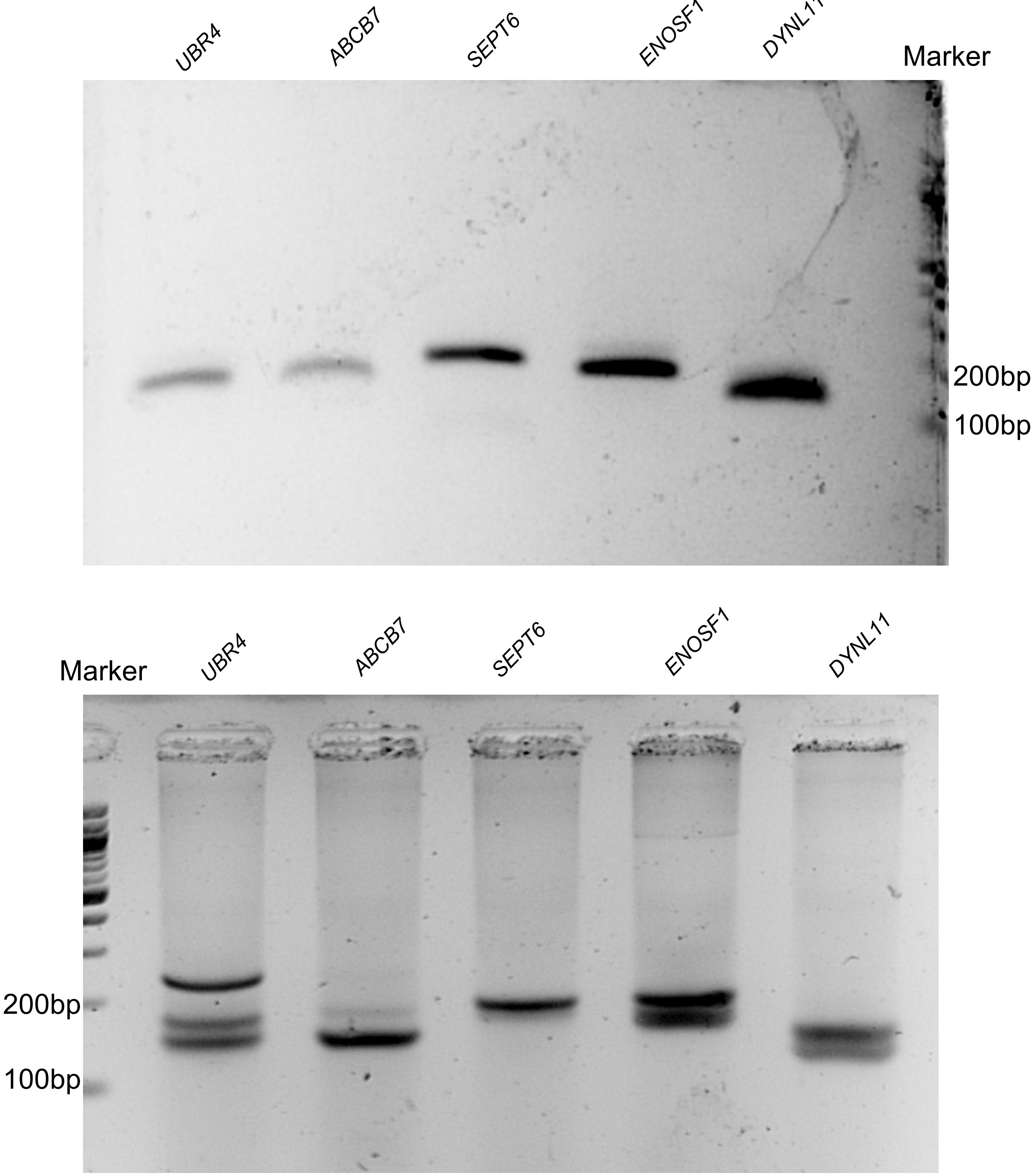
UBR4
ABCB7
SEPT6
ENOSF1
DYNL11
Marker
200bp
100bp
Marker
UBR4
ABCB7
SEPT6
ENOSF1
DYNL11
200bp
100bp

Validation of cryptic 3’ splice site usage in five selected genes frequently mis spliced in *SF3B1* mutant myelodysplastic syndrome. PCR products of the five genes (*UBR4, ABCB7, SEPT6, ENOSF1, DYNL11*) showing aberrant cryptic 3’SS usage in the K562 *SF3B1* mutant (bottom panel) as opposed to the WT K562 (top panel). The top and bottom panels correspond to the left and right halves of Figure 4c
